# Validity and Reliability of Upper Limb Functional Assessment Using the Microsoft Kinect V2 Sensor

**DOI:** 10.1155/2019/7175240

**Published:** 2019-02-11

**Authors:** Laisi Cai, Ye Ma, Shuping Xiong, Yanxin Zhang

**Affiliations:** ^1^The Research Academy of Grand Health, Faculty of Sport Science, Ningbo University, Ningbo, China; ^2^Department of Industrial and Systems Engineering, College of Engineering, Korea Advanced Institute of Science and Technology (KAIST), Republic of Korea; ^3^Department of Exercise Science, Faculty of Science, The University of Auckland, New Zealand

## Abstract

**Objective:**

To quantify the concurrent accuracy and the test-retest reliability of a Kinect V2-based upper limb functional assessment system.

**Approach:**

Ten healthy males performed a series of upper limb movements, which were measured concurrently with Kinect V2 and the Vicon motion capture system (gold standard). Each participant attended two testing sessions, seven days apart. Four tasks were performed including hand to contralateral shoulder, hand to mouth, combing hair, and hand to back pocket. Upper limb kinematics were calculated using our developed kinematic model and the UWA model for Kinect V2 and Vicon. The interdevice coefficient of multiple correlation (CMC) and the root mean squared error (RMSE) were used to evaluate the validity of the kinematic waveforms. Mean absolute bias and Pearson's *r* correlation were used to evaluate the validity of the angles at the points of target achieved (PTA) and the range of motion (ROM). The intersession CMC and RMSE and the intraclass correlation coefficient (ICC) were used to assess the test-retest reliability of Kinect V2.

**Main Results:**

Both validity and reliability are found to be task-dependent and plane-dependent. Kinect V2 had good accuracy in measuring shoulder and elbow flexion/extension angular waveforms (CMC > 0.87), moderate accuracy of measuring shoulder adduction/abduction angular waveforms (CMC = 0.69-0.82), and poor accuracy of measuring shoulder internal/external angles (CMC < 0.6). We also found high test-retest reliability of Kinect V2 in most of the upper limb angular waveforms (CMC = 0.75-0.99), angles at the PTA (ICC = 0.65-0.91), and the ROM (ICC = 0.68-0.96).

**Significance:**

Kinect V2 has great potential as a low-cost, easy implemented device for assessing upper limb angular waveforms when performing functional tasks. The system is suitable for assessing relative within-person change in upper limb motions over time, such as disease progression or improvement due to intervention.

## 1. Introduction

Three-dimensional (3D) upper limb functional movements such as reaching, pushing/pulling, and throwing have been studied in many areas including motor control [1, 2], neurophysiology [3], clinical assessment and rehabilitation [4–6], and ergonomics [7, 8]. Currently, quantitative measurements of upper limb functions are normally carried out using marker-based motion capture systems [9], in which the 3D motion data is obtained based on the passive or active markers attached on the anatomical landmarks of participants. Although the marker-based systems in assessing upper limb kinematics [3, 5] have been confirmed to be valid and reliable, these systems require relatively large spaces, are expensive, and require experienced technicians, therefore limiting their use in the clinic, at home, in public, and so forth. In comparison, a markerless motion capture system would be a possible alternative [10] for upper limb assessment.

Microsoft Kinect is a low-cost markerless motion capture system, which estimates the 3D location of body joints based on 2D images with depth information using machine learning algorithms [11]. Kinect is feasible to assess gait temporal-spatial parameters and kinematics [12] and can objectively evaluate static foot posture with good accuracy and reliability [13]. Kinect has the potential to be used as an assessment tool for certain aspects of the balance performance [14]. Kinect can also assist gait rehabilitation training in clinics by providing the lateral trunk lean angle as a real-time feedback [15]. Some research investigated the use of Kinect in clinics [4] and confirmed that Kinect can accurately measure gross spatial characteristics such as lower limb and trunk kinematics but cannot measure smaller movements such as hand clapping with the same accuracy. Researchers also investigate the use of Kinect in the workplace and found that Kinect can determine risks of musculoskeletal injuries in the workplace [16].

Attempts have been made at using Kinect in upper limb assessment [17–19]. Different scales have been used as outcome measures for disease progressions and medical interventions, which are subjective and could vary depending on different observers. Therefore, quantitative data attained by measuring kinematics is necessary for therapy practice. Chen et al. [17] developed a Kinect-based system to measure active upper limb movements as a complementary output measure of functional rating scales for spinal muscular atrophy. They observed no significant differences in the active range of motion (ROM) between the patients and the controls. They also found that the Kinect-based system is not sensitive enough to capture the minor differences or early-stage progression in the high-functioning patient group [17]. Moreira et al. [18] developed a Kinect-based system for upper body function assessment in breast cancer patients. Based on the extracted upper limb kinematic features, the Kinect-based classification system can diagnose upper limb impairments for breast cancer patients. Kinect has also been used to assess 3D shoulder kinematics during computer use to provide some insight on shoulder kinematics for improving office ergonomics [19].

Establishing the accuracy and reliability inherent in the Microsoft Kinect system is required before using it for upper limb assessment. The accuracy of the Kinect system in measuring lower limb kinematics has been evaluated using marker-based measurements as the gold standard [4, 12, 20, 21]. The reliability of the Kinect measurement has also been studied in postural control assessment [21], gait analysis [12], and static foot posture evaluation [13]. However, to the best of our knowledge, a thorough validity and reliability study of the Kinect system on assessing 3D upper limb kinematics when performing functional tasks is lacking. The goal of this study was to quantify the accuracy and test-retest reliability of a Kinect motion capture system in assessing upper limb kinematics when performing functional tasks. A marker-based motion capture system (Vicon, Oxford Metrics Group, Oxford, UK) was used as the gold standard measurement.

## 2. Methodology

### 2.1. Subjects

Ten healthy male university students (age: 24.6 ± 2.8 years, height: 174.05 ± 4.4 cm, mass: 67.2 ± 4.2 kg) with no upper limb injuries or medication use that would have influenced their upper limb functions volunteered to participate. Participants were informed about the basic procedure of the experiment before the test. The experimental protocol was approved by the Research Academy of Grand Health's Ethics Committee at Ningbo University.

### 2.2. Testing Procedure

This study used a concurrent validity, test-retest reliability design. The study was conducted at the biomechanics laboratory of Ningbo University. Upper limb kinematics were recorded concurrently by a Kinect V2 system with a sampling frequency of 30 Hz and a 3D motion capture system with eight infrared high-speed cameras (Vicon, Oxford Metrics Ltd., Oxford, UK) with a sampling frequency of 100 Hz. Prior to data collection, Kinect V2 was placed on a tripod at 0.8 meters above the floor. Subjects stood at 2 meters from the camera according to the recommendation [16].

Each participant attended two testing sessions, seven days apart. For each session, reflective markers were attached to the anatomical landmarks of the participants according to the UWA upper limb model [22]. First, a static trial is performed during which each participant stands in the anatomical position. Then, the elbow and wrist markers were removed during the following dynamic trials. Four functional tasks were performed which represent a range of functional activities [23].

**Figure 1:**
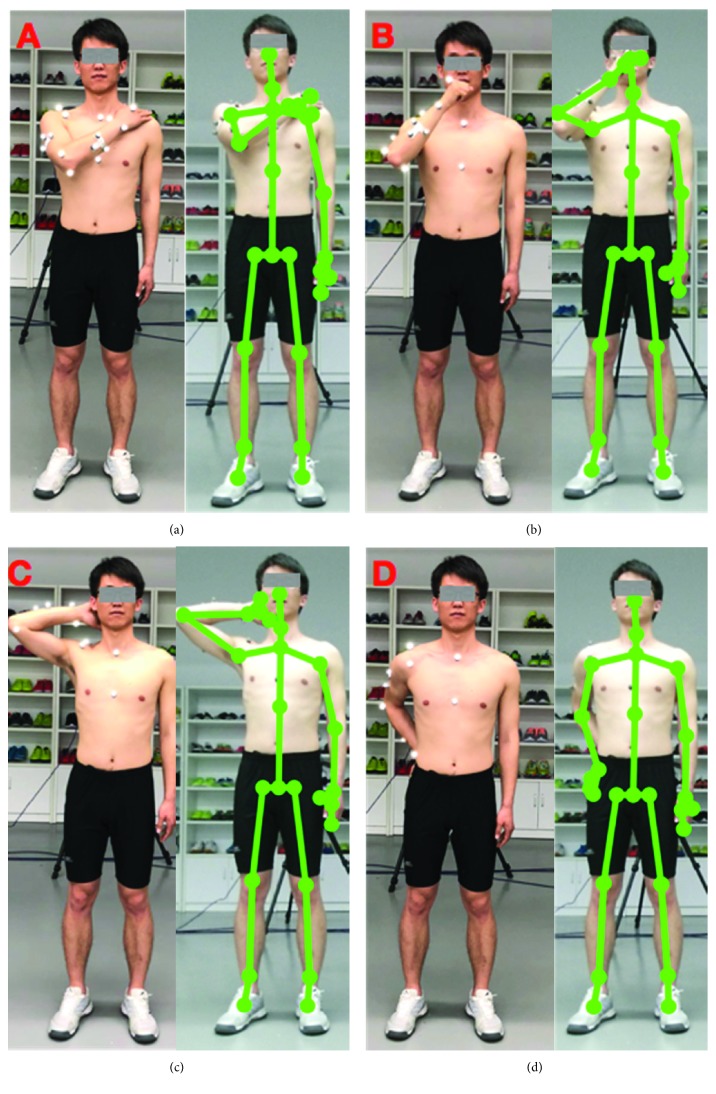
Four upper limb functional tasks performed in the study.

- Task one is hand to the contralateral shoulder, which represents all activities near contralateral shoulder such as washing axilla or zip up a jacket. Subjects started with the arm in the anatomical position with their hand beside their body in a relaxed neutral position and end up with the hand touching the contralateral shoulder (see Figure 1(a))
- Task two is hand to mouth or drinking, which represents activities such as eating and reaching the face. It begins with the same starting point and ends when the hand reached the subject's mouth (see Figure 1(b))
- Task three is combing hair, which represents activities such as reaching the (back of the) head and washing hair. Subjects were instructed to move their hand to the back of their head (see Figure 1(c))
- Task four is hand to back pocket, which represents reaching the back and perineal care. It begins with the same starting point and ends when the hand is placed on the back pocket (see Figure 1(d))

At least five trials were collected for each task.

### 2.3. Upper Limb Models for the Vicon System and the Kinect V2 System

**Figure 2:**
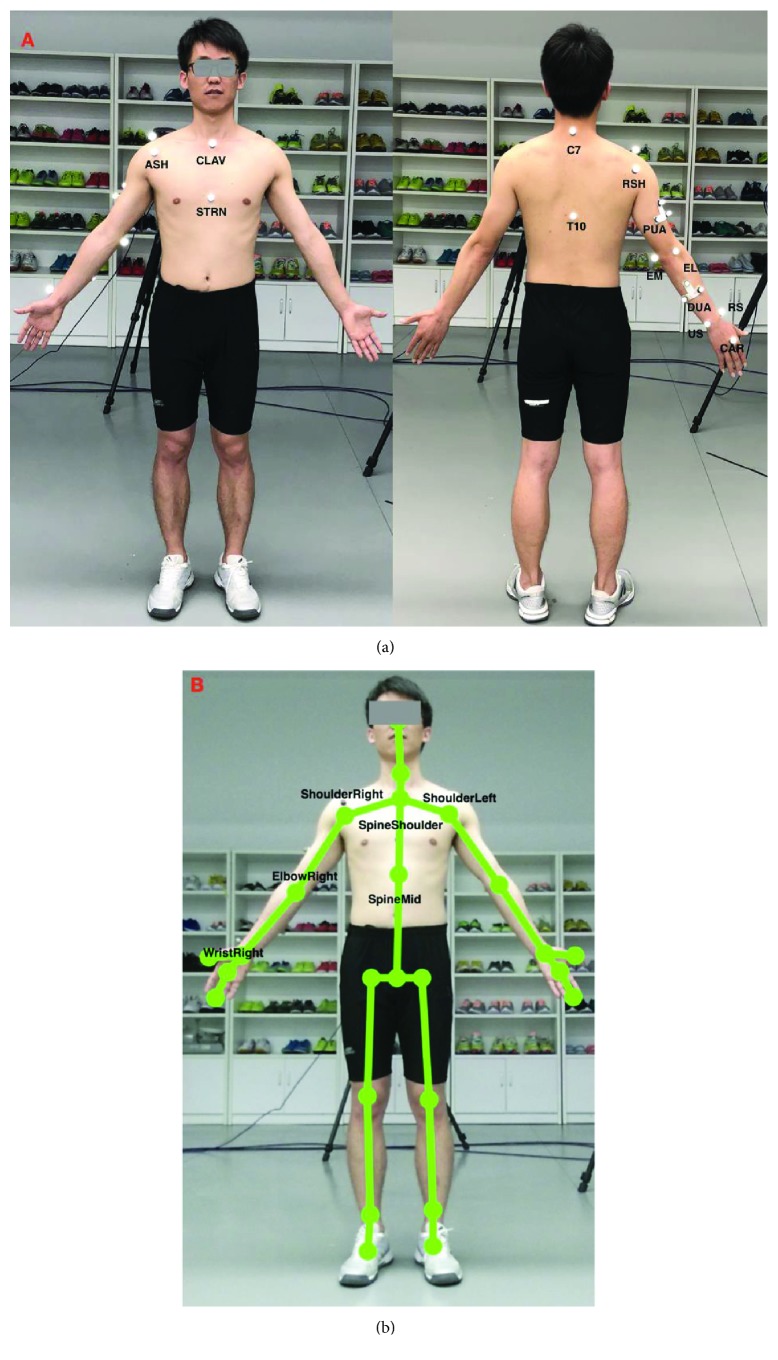
The upper limb models for the Vicon system and the Kinect V2 system ((a) the upper body marker set for the Vicon system; (b) the skeleton model of the Kinect V2 system).

The Vicon system tracked and stored the spatial trajectories of the reflective markers attached to the subjects. The UWA upper limb marker set was employed in this study [24], which includes 18 markers (see Figure 2). Trunk, upper arm, forearm, and hand segments were defined based on the anatomical landmark positions. The definition of the upper limb segment coordination system for the Vicon system is presented in Table 1. The calibrated anatomical systems technique [25] is used to establish the motion of anatomical landmarks relative to the coordinate systems of the upper-arm cluster (PUA) or the forearm cluster (DUA). The motion of the upper limb landmarks could be reconstructed from their constant relative positions to the upper-arm technical coordinate system. The kinematic model based on the UWA upper limb model was developed using Vicon Bodybuilder software. The reference shoulder and elbow joint angles were calculated based on the measured position of the passive optical markers using the kinematic model via Vicon Nexus software (Oxford Metrics Group, Oxford, UK). A Butterworth low-pass filter was used with the cut-off frequency of 6 Hz for both Vicon and Kinect V2 systems. For a more detailed description, see [22, 24].

The 3D coordinates of the anatomical landmarks identified from the skeletal model of the Kinect V2 system during functional tasks were also recorded. Local segment coordinates including torso and upper arm were established, and each of them was based on the global coordinate (Table 2). Then, our customized upper limb kinematics for the Kinect V2 system calculated the three Euler angles for shoulder rotations, which follows the flexion (+)/extension (-), adduction (+)/abduction (-), and internal (+)/external (-) rotation order (see Figure 2). The elbow flexion was calculated by the position data from ShoulderRight, ElbowRight, and WristRight using the trigonometric function. The kinematics model for Kinect V2 was developed using Matlab 2017a. The angular waveforms between the Kinect V2 sensor and the Vicon system were synchronized during post-processing. The joint angles from both systems were firstly resampled to 300 Hz using the Matlab function “interp” and then synchronized using a cross-correlation-based shift synchronization technique.

### 2.4. Statistical Analysis

The concurrent validity of the Kinect V2 system for assessing the upper limb functional movement waveforms was carried out using the coefficient of multiple correlation (CMC) [26] and the root mean squared error (RMSE) between the waveforms calculated by the Kinect V2 and the Vicon-based system. Mean bias (Kinect-Vicon) and Pearson's *r* correlation between the two systems were used to evaluate the concurrent validity of the Kinect-based system in assessing the joint angles at the point of target achieved (PTA) and the range of motion (ROM). Paired Student *t*-tests were used to compare the results of the angles at the PTA and the ROM with the significant level of 0.01. The concurrent validity was presented using data from session one.

The CMC and RMSE between the waveforms from session one and session two measured by the Kinect V2 system were used to assess the relative and absolute test-retest reliability. The reliability of the Kinect V2 system in assessing the joint angles at the PTA and the ROM from selected functional tasks was also carried out using the intraclass correlation (ICC_3,*k*_) coefficient with the absolute measure. The ICC and Pearson's *r* correlation as well as the descriptive statistics were performed using SPSS 22.0. The CMC and RMSE were analyzed using Matlab 2017a.

## 3. Results

### 3.1. The Concurrent Validity of the Kinect V2 System for Upper Limb Functional Assessment

**Figure 3:**
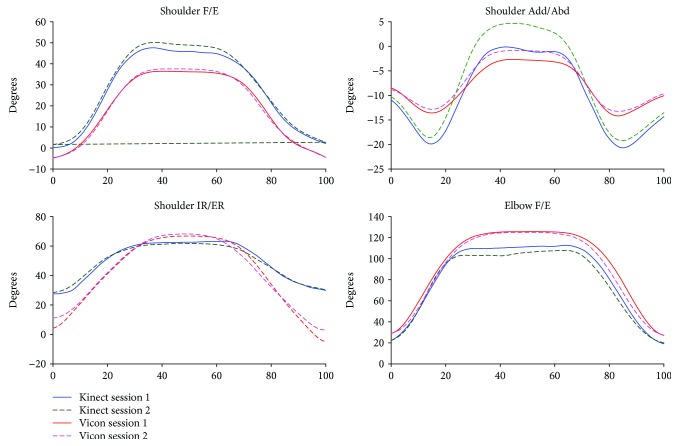
Kinematics from hand to contralateral shoulder task.

**Figure 4:**
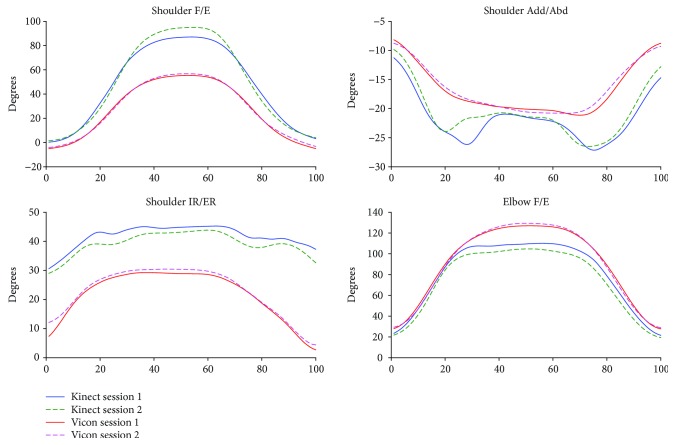
Kinematics from hand to mouth/drinking task.

**Figure 5:**
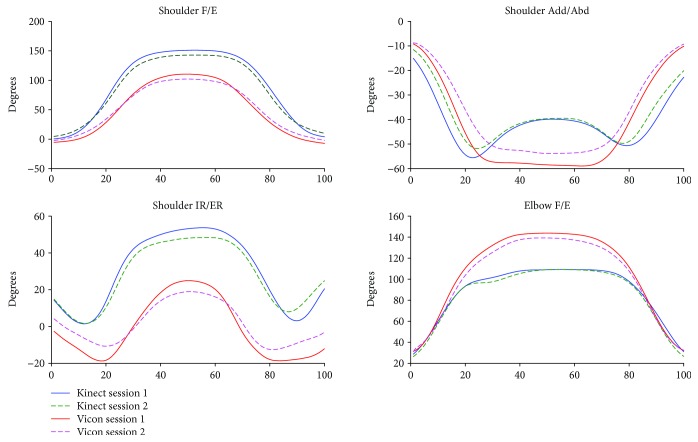
Kinematics from combing hair task.

**Figure 6:**
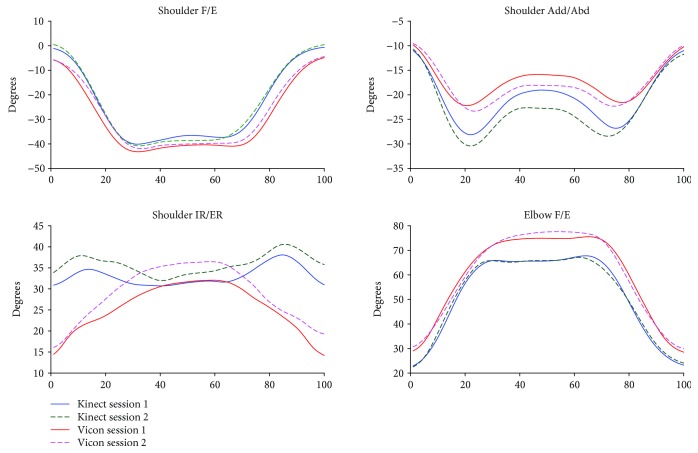
Kinematics from hand to back pocket task.

The kinematic waveforms of the selected upper limb functional tasks in both sessions from the Kinect V2 system and the Vicon system are presented in Figures 3-6 by means of the average segment rotation angles. The validity of the Kinect V2 system in assessing upper limb angular waveforms, joint angles at the PTA, and the ROM are presented in Tables 3 and 4.

High-level agreements (see Table 3) were observed for shoulder and elbow kinematics in the sagittal plane across all tasks with the CMC values of 0.81-0.94. Compared to the shoulder joint, elbow flexion/extension angles showed the best agreements between the two systems with the CMC greater than 0.9 for all tasks except for the combing hair task (CMC = 0.87). Shoulder adduction/abduction angular waveforms showed moderate agreements between the two systems with the CMC values of 0.69-0.82. The lowest CMCs between the two systems were found in the transverse plane at the shoulder joint with the CMC values of lower than 0.6 except for the hand to contralateral shoulder task (CMC = 0.84).

The RMSEs (see Table 3) between the angular waveforms from the two systems are also both plane-dependent and task-dependent. For angular waveforms in the sagittal plane at shoulder and elbow joint, the lowest RMSEs were found in the hand to back pocket task with the RMSEs of shoulder and elbow flexion/extension angles of 7.16° and 10.43° and the highest RMSEs were identified in the combing hair task with the RMSEs of 41.4° and 23.75° for shoulder flexion/extension elbow flexion/extension angles. For angular waveforms in the frontal plane at the shoulder joint, i.e., the shoulder adduction/abduction angle, the RMSEs between the Kinect V2 and Vicon systems are under six degrees except for the combing hair task (RMSE = 12.31°). For shoulder angular waveforms in the transverse plane, i.e., the shoulder internal/external rotation angle, the smallest RMSE was found in the hand to mouth drinking task (RMSE = 1.64°) and the biggest RMSE was found in the combing hair task (RMSE = 29.38°).

Mean (SD) values for joint angles at the PTA and the ROM estimated by the Kinect V2 and Vicon systems are provided in Table 4. Excellent relative agreements (*r* = 0.73–0.97) were observed for all investigated angles at PTA in all tasks except for moderate relative agreement of the shoulder internal/external rotation in the hand to back pocket task and elbow flexion/extension in the hand to contralateral shoulder task (*r* = 0.46 and *r* = 0.45, respectively) and poor agreement of the shoulder and elbow flexion/extension angle in the combing hair task (*r* = −0.20 and *r* = 0.21, respectively). For the ROM, excellent relative agreements were observed for shoulder flexion/extension and shoulder adduction/abduction angles in all tasks (*r* = 0.91-0.99) except for the combing hair task (*r* = 0.20 and 0.65, respectively); excellent agreements were found for shoulder internal/external rotation angles only in the shoulder to contralateral shoulder task and the combing hair task (*r* = 0.74 and 0.77), respectively; poor to moderate agreements were found for elbow flexion/extension angles in all tasks (*r* < 0.65) except for the hand to back pocket task, which showed excellent agreement (*r* = 0.97).

There is a clear tendency that the Kinect V2 system overestimated shoulder flexion/extension angles and underestimated elbow flexion/extension angles in all tasks. According to the mean absolute bias of the angles at the PTA between the Kinect V2 and Vicon systems (see Table 2, K-V), there were no significant bias only for shoulder flexion/extension, shoulder internal/external rotation, and elbow flexion/extension angles in the hand to back pocket task and shoulder adduction/abduction angle in the hand to mouth/drinking task. For the absolute bias of the ROM, only elbow flexion/extension in the first and last tasks, the shoulder adduction/abduction angle in the second task, and the shoulder internal/external rotation in the second and fourth tasks showed no significant differences. The greatest biases of the ROMs were found in the shoulder flexion/extension angles in the hand to mouth/drinking task and the combing hair task (RMSE = 32.5-40.9°).

### 3.2. The Test-Retest Reliability of the Kinect V2 System for Upper Limb Functional Assessment

The test-retest reliability of the Kinect V2 system in assessing the upper limb angular waveforms, the angles at the PTA, and the corresponding ROM is presented in Table 5. Good to excellent relative reliability (CMC = 0.75-0.99) was observed for all between-session angular waveforms across all selected tasks except for the shoulder adduction/abduction in task one (CMC = 0.7) and the shoulder internal/external rotation in the fourth task (CMC = 0.6). Angular waveforms in the sagittal plane (CMC = 0.89-0.99) are more reliable than those in the frontal plane (0.70-0.84) and transverse plane (CMC = 0.60-0.93). The RMSEs are under ten degrees except for elbow F/E (RMSE = 8.3-11.03) in the first three tasks and shoulder IR/ER in task 3 (RMSE = 12.09). The worst absolute test-retest reliability was observed in the combing hair task, in which the RMSE of shoulder flexion/extension is the biggest (18.91°).

Results for the test-retest reliability of the Kinect V2 system in assessing the angles at the PTA and the ROM are also presented in Table 5. There are no significant between-session differences in the Kinect V2 system for all investigated angles for all of the studied motions. All between-session ICCs of the angle at the PTA from all motions are good to excellent for the Kinect V2 system (ICC = 0.65-0.91) in all selected tasks except for shoulder adduction/abduction in the first task (ICC = 0.59) and elbow flexion/extension in the combing hair task (ICC = 0.27). All between-session ICCs of the ROM measurement from all studied motions are good to excellent for the Kinect V2 system (ICC = 0.68-0.96) except for the motions in the combing hair task (ICC = 0.35-0.70). The lowest reliability of the ROM was shoulder flexion/extension in the combing hair task with the mean ICC of 0.35.

## 4. Discussion

This study tested the concurrent validity and the test-retest reliability of upper limb functional assessment using Kinect V2. We found that both validity and reliability are task-dependent and plane-dependent.

The Kinect V2 system had good accuracy in measuring shoulder and elbow flexion/extension angles, moderate accuracy of measuring shoulder adduction/abduction angles, and poor accuracy of measuring shoulder internal/external angles. We also found high test-retest reliability of the Kinect V2 system in most of the upper limb angular waveforms, angles at the PTA, and the corresponding ROM. However, there are also some deviations both between the Kinect V2 system and the Vicon system (gold standard) and between two test sessions.

### 4.1. Concurrent Validity of Kinect V2

The angular trajectories from the Kinect V2 and Vicon have similar waveform patterns, especially for the flexion/extension angular waveforms under all functional tasks (CMC = 0.81-0.94). Nevertheless, the RMSEs between the kinematic patterns measured by the Kinect V2 system and Vicon system are not consistent with the angular waveform agreements.

Generally, the mean deviations between joint angles assessed by the Kinect V2 system and the Vicon system are greater for tasks or planes with a larger range of motions. The shoulder and elbow angular waveforms in the sagittal plane measured by the Kinect V2 system highly agreed with the reference angles, and the RMSEs of the Kinect V2 system in measuring sagittal plane kinematics are generally greater or comparable than angles in the frontal plane and transverse plane in comparison with those from the Vicon system. Similarly, the accuracy of the Kinect V2 system in assessing angles at the PTA and the ROM are task-dependent and plane-dependent. The angles at the PTA showed good relative agreement for almost all motions investigated except for motions in the combing hair task, which have a large range of motion.

Our finding agrees with the results of [19], which found that the Kinect V2 sensor had a better estimation on shoulder flexion/extension, compared with shoulder adduction/abduction and shoulder internal/external rotation during the computer operation tasks [19]. They also found that the shoulder flexion/extension angle had the lowest RMSE (under 15°) and the magnitude of error is proportional to the magnitude of the shoulder adduction/abduction angle [19]. The concurrent validity results of the upper limb motions are not as good as those for lower limb and trunk motions.

The measurement performance of the Kinect V2 system is highly varied on tasks, joints, and planes of movement. According to Clark et al.'s study [15, 21], the Kinect V2 system showed excellent concurrent validity with the Vicon system, with the Pearson *r* values >0.9 for the majority of measurements for trunk and lower limb motions during lateral reach, forward reach, and single leg balance. In contrast, the Kinect V2 system showed lower concurrent validity in assessing upper limb motions in our study with the Pearson *r* values of 0.7-0.99 for most upper limb motions at the PTA and the ROM.

The abovementioned findings can be explained by the underlying real-time human pose recognition algorithm and the nature of the single-depth camera. The Kinect system estimates the 3D location of body joints based on 2D images with depth information using machine learning algorithms. The final set of confidence-weighted 3D joint proposals is based on a global optimization algorithm using training data, which represents postures in an entertainment environment [11]. Therefore, Kinect should be evaluated carefully before employing it as a research tool. If a joint is shaded by other body parts, it is difficult for the Kinect system to define the corresponding anatomical landmarks of the Kinect skeletal model, which directly results in inaccurate joint angle prediction. Anatomical landmarks of the trunk and lower limb identified from the skeletal model of the Kinect system may be more accurate because the segments of the trunk and lower limb are usually not shaded by other body parts during the tasks in reach, balance test, or walking. This may be the main reason for the higher validity in the assessment of trunk and lower limb movements in comparison with those of upper limb tasks.

### 4.2. Test-Retest Reliability of Kinect V2

We found a high degree of reproducibility in almost all of the upper limb angular waveforms, angles at the PTA, and the ROM measured by the Kinect V2 system during all functional tasks across the two testing sessions. Our finding suggests that the Kinect V2 system can reliably assess 3D shoulder motions and elbow flexion/extension for individuals carrying out such functional tasks. The repeatability of upper limb motions in the frontal and transverse planes was lower than that in the sagittal plane. However, there were still some between-session deviations in the starting point and ending point of the upper limb. Greater standardization of both the starting and ending points for the tasks would be required to improve the repeatability of the starting and ending points of this type of motion. The test-retest reliability of the trunk motion is high (ICC > 0.73) during lateral reach and forward reach in Clark et al.'s research [15], which is similar to results of the upper limb motions in our study.

The combing hair task is not an ideal upper limb functional task when using the Kinect V2 system as the outcome measure tool. During the combing hair task, the Kinect V2 system had the worst accuracy and reliability in assessing the angular waveforms, the angles at the PTA, and the corresponding ROM. The greatest deviations of the angular waveforms for the Kinect V2 system were identified for all investigated motions in the combing hair task. The Kinect V2 system also cannot measure shoulder and elbow flexion/extension angles at the PTA and the ROMs of all investigated motions in the combing hair task with low test-retest reliability (ICC = 0.27-0.65). During the task, the absolute test-retest reliability of shoulder F/E is the worst with the between-session RMSE of 18.91°. Thus, it is better not to use the same functional task like combing hair in the assessment of upper limb functions.

### 4.3. Clinical Implications

Our results show that the Kinect V2 system can reliably measure upper limb motions. Kinect V2 has good relative agreements of angular waveforms and can accurately measure a few of the shoulder and elbow joint angles during the functional tasks. Although the measurement of upper limb kinematics may be not as accurate as the Vicon system, Kinect V2 is useable to track relative within-person changes in movements over time (such as the worsening of movement symptoms with disease progression or improvement due to intervention).

In current clinical practice, clinical scales have been widely used to assess upper limb functions, which are subjective and labour-intensive. The development of a video tracking system based on low-cost markerless cameras would free clinicians from the necessity of dedicated (and expensive) motion capture laboratories and would allow for in situ data collection. This markerless system would have a significant impact in the fields of clinical practice. Unlike clinical scales, the markerless motion analysis technique is objective, which helps to quantify massive kinematic parameters and turn data into a knowledge-based data warehouse in a standard way. The whole process makes it possible for identifying and explaining relationships between different motion patterns or different population groups through data mining.

### 4.4. Limitation and Future Work

The experiment had a limited sample size (ten male university students). The system has not yet been used in other populations or other environments such as upper limb disordered patients in clinical settings, elderly people at home or in the clinical environment, and people working in the workplace. The finding from this study may have differed if the assessment was undertaken in a clinical population. Upper limb motions of healthy people often have low variability. The low variability increases heterogeneity and would potentially lead to higher reliability estimates and stronger correlations between Kinect V2 and the reference. In the future, tests with larger sample size, including patients, will be conducted.

Further work is also concerned to improve the accuracy of the Kinect system in measuring upper limb joint angles. There have been several recent studies in estimating body landmarks and motions using the Kinect sensor [27, 28]. It is likely that using these techniques would possibly produce more accurate joint kinematics. Attempts have also been made using multiple Kinect sensors concurrently to improve the accuracy of tracking movement [28]. Calibration algorithms are another solution to improve the joint angle prediction accuracy of the Kinect system. Xu and colleagues used the linear regression algorithm to calibrate the shoulder adduction/abduction angle, and the prediction accuracy was significantly improved [19]. Using the state-of-the-art machine learning algorithms, it is possible to improve the current joint kinematic measurement accuracy as high agreements were found for angles in the sagittal and frontal planes (CMC > 0.78) for most investigated motions.

## 5. Conclusion

The Kinect V2-based upper limb functional assessment system developed in this research has good test-retest reliability in assessing the upper limb angular waveforms and the angles at the point of target achieved except for the combing hair task. The Kinect V2-based system can accurately assess shoulder flexion/extension, elbow flexion/extension, and shoulder adduction/abduction in some upper limb functional tasks. The Kinect V2 sensor has great potential as a low-cost, easily implemented device for assessing upper limb angular waveforms when performing functional tasks. Our system is suitable for assessing relative within-person change in upper limb motions over time, such as disease progression or improvement due to intervention.

## Figures and Tables

**Table 1:** The upper arm and torso anatomical segment coordinate systems for the Vicon system.

Name		Definition
Torso	Origin	C7
	X	Unit vector defined by the *Y*-axis and the *Z*-axis to create a right-hand coordinate system
	Y	Unit vector going from T10 to C7
	Z	Unit vector perpendicular to the sagittal plane defined by T10, C7, and CLAV, pointing laterally

Right upper arm	Origin	The elbow joint center, which was the midpoint between EL and EM
	X	Unit vector perpendicular to the *Y*-axis and the *Z*-axis, pointing anteriorly
	Y	Unit vector going from the elbow joint center to the shoulder joint center (the midpoint between ASH and PSH)
	Z	Unit vector perpendicular to the plane formed by the *Y*-axis of the upper arm and the long-axis vector of the forearm.

Note: C7: 7th cervical vertebra; CLAV: clavicular notch; EC: elbow center; EL: lateral epicondyle; EM: medial epicondyle; PSH: posterior shoulder; RS: radial styloid; STRN: sternum; T10: 10th thoracic vertebra; US: ulnar styloid.

**Table 2:** The upper arm and torso anatomical segment coordinate systems for the Kinect V2 system.

Name		Definition
Torso	Origin	SpineShoulder
	X	Unit vector perpendicular to two vectors (Y and the vector from ShoulderRight to ShoulderLeft)
	Y	Unit vector going from SpineMid to SpineShoulder
	Z	Unit vector defined by the *X*-axis and the *Y*-axis to create a right-hand coordinate system

Right upper arm	Origin	The elbow joint center (ElbowRight)
	X	Unit vector perpendicular to the *Y*-axis and the *Z*-axis, pointing anteriorly
	Y	Unit vector going from the elbow joint center to the shoulder joint center (ElbowRight to ShoulderRight)
	Z	Unit vector perpendicular to the plane formed by the *Y*-axis of the upper arm and the long-axis vector of the forearm

**Table 3:** Validity of the angular waveforms for the selected functional tasks. The mean (SD) coefficient of multiple correlation (CMC) and the root mean squared error (RMSE) between the Kinect V2-based markerless system and the Vicon-based system of the upper limb waveforms are presented.

	Hand to contralateral shoulder				Hand to mouth/drinking				Combing hair				Hand to back pocket			
	Shoulder F/E	Shoulder Add/Abd	Shoulder IR/ER	Elbow F/E	Shoulder F/E	Shoulder Add/Abd	Shoulder IR/ER	Elbow F/E	Shoulder F/E	Shoulder Add/Abd	Shoulder IR/ER	Elbow F/E	Shoulder F/E	Shoulder Add/Abd	Shoulder IR/ER	Elbow F/E
CMC	0.88 (0.09)	0.82 (0.11)	0.84 (0.07)	0.94 (0.05)	0.82 (0.09)	0.80 (0.2)	0.58 (0.20)	0.92 (0.04)	0.81 (0.11)	0.78 (0.13)	0.54 (0.17)	0.87 (0.05)	0.93 (0.05)	0.69 (0.27)	0.55 (0.27)	0.90 (0.13)
RMSE	11.04 (5.16)	5.62 (1.37)	13.40 (3.00)	15.48 (6.04)	25.61 (7.56)	6.11 (2.85)	1.64 (5.20)	18.16 (4.95)	41.40 (9.49)	12.31 (2.65)	29.38 (6.76)	23.75 (4.32)	7.16 (2.87)	5.76 (2.76)	12.36 (3.74)	10.43 (6.21)

Note: shoulder F/E, shoulder Add/Abd, shoulder IR/ER, and elbow F/E represent joint angles of shoulder flexion/extension, shoulder adduction/abduction, shoulder internal/external rotation, and elbow flexion, respectively.

**Table 4:** Concurrent validity of the joint angles at the point of target achieved (PTA) and the ROM of the selected functional tasks. The mean (SD) peak joint angles and the ROM are presented with the Pearson correlation (*r*) and the discrepancy (Kinect-Vicon) between the Kinect V2-based markerless system and the Vicon-based system is presented.

Segment rotations	PTA					ROM				
	Kinect	Vicon	P_(K,V)_	Bias (K-V)	*r*	Kinect	Vicon	P_(K,V)_	Bias (K-V)	*r*
*Shoulder to contralateral shoulder*										
Shoulder F/E	52.1 (10.3)	38.1 (6.7)	<0.01	14.0	0.74	51.5 (10.0)	43.7 (6.9)	<0.01	7.8	0.98
Shoulder Add/Abd	5.5 (7.8)	−0.4 (6.3)	<0.01	5.9	0.89	26.2 (5.8)	14.3 (3.9)	<0.01	7.9	0.99
Shoulder IR/ER	63.7 (6.8)	68.5 (5.7)	<0.01	−4.8	0.89	38.7 (11.7)	66.7 (14.9)	<0.01	−28	0.74
Elbow F/E	110.9 (8.7)	125.2 (3.5)	<0.01	−14.3	0.45	91.8 (8.3)	98.2 (6.2)	0.06	−6.4	0.41
*Hand to mouth/drinking*										
Shoulder F/E	95.6 (19.4)	57.3 (10.2)	<0.01	38.3	0.88	94.8 (19.4)	62.3 (12)	<0.01	32.5	0.91
Shoulder Add/Abd	−8.5 (4.5)	−8.2 (2.5)	0.67	−0.3	0.85	20.4 (9.6)	14.4 (13.4)	<0.01	6.0	0.95
Shoulder IR/ER	47.5 (11.4)	32.3 (7.3)	<0.01	15.2	0.93	21.7 (9)	29.2 (10.5)	0.07	−7.5	0.33
Elbow F/E	107.1 (9.3)	129.6 (5.7)	<0.01	−22.5	0.80	89.1 (10.1)	102.3 (6.6)	<0.01	−13.2	0.65
*Combing hair*										
Shoulder F/E	143.5 (14.1)	102.6 (20.4)	<0.01	40.9	−0.20	140 (22.7)	106 (15.6)	<0.01	34.0	0.20
Shoulder Add/Abd	−53.7 (7.6)	−54.7 (7.2)	0.40	1.0	0.89	42.2 (6.4)	46.9 (5.6)	0.02	−4.7	0.65
Shoulder IR/ER	49.4 (11.2)	25.3 (10.2)	<0.01	24.1	0.72	55.6 (16.9)	42.8 (10.9)	<0.01	12.8	0.77
Elbow F/E	113.6 (3.4)	139.6 (14.1)	<0.01	−26	0.21	91.1 (7.7)	110.9 (13.7)	<0.01	−19.8	0.05
*Hand to back pocket*										
Shoulder F/E	−41.8 (6.2)	−42.9 (6.1)	0.47	1.1	0.73	43.4 (6.7)	38.7 (6.1)	<0.01	4.7	0.91
Shoulder Add/Abd	−33 (10.3)	−25.5 (7.6)	<0.01	−7.5	0.97	22.9 (9.6)	16.7 (7.6)	<0.01	6.2	0.96
Shoulder IR/ER	42.3 (9.6)	37.7 (5.7)	0.02	4.6	0.46	18.6 (7.9)	23.3 (5)	0.16	−4.7	−0.10
Elbow F/E	73.7 (21.3)	81.2 (13.2)	0.03	−7.5	0.94	51.8 (25)	52.1 (18.1)	0.91	−0.3	0.97

Note: max and min are the maximum and minimum joint angles, respectively. ROM: corresponding range of motion. Shoulder F/E, shoulder Add/Abd, shoulder IR/ER, and elbow F/E represent joint angles of shoulder flexion/extension, shoulder adduction/abduction, shoulder internal/external rotation and elbow flexion, respectively; K-V is the bias between the Kinect V2 and Vicon systems.

**Table 5:** Reliability of the angular waveforms, the angles at the point of target achieved (PTA), and the range of motion (ROM) for the selected functional tasks. The mean (SD) coefficient of multiple correlation (CMC) and the root mean squared error (RMSE) of the upper limb waveforms calculated by the Kinect V2 system between two sessions are presented. The intraclass correlation (ICC) of the angles at the PTA and the ROM of the selected functional tasks are also presented.

	Segment rotation	CMC	RMSE	ICC	
				PTA	ROM
Hand to contralateral shoulder	Shoulder F/E	0.93 (0.08)	7.89 (3.88)	0.80	0.88
	Shoulder Add/Abd	0.70 (0.28)	9.19 (5.29)	0.59	0.68
	Shoulder IR/ER	0.93 (0.06)	5.93 (1.87)	0.88	0.96
	Elbow F/E	0.97 (0.3)	11.03 (4.50)	0.86	0.80

Hand to mouth/drinking	Shoulder F/E	0.99 (0.09)	9.90 (8.67)	0.80	0.81
	Shoulder Add/Abd	0.75 (0.28)	5.03 (2.98)	0.78	0.95
	Shoulder IR/ER	0.75 (0.28)	7.25 (5.64)	0.76	0.80
	Elbow F/E	0.96 (0.05)	10.70 (5.77)	0.72	0.84

Combing hair	Shoulder F/E	0.97 (0.03)	18.91 (8.11)	0.65	0.35
	Shoulder Add/Abd	0.84 (0.12)	7.70 (3.69)	0.65	0.43
	Shoulder IR/ER	0.90 (0.06)	12.09 (4.52)	0.83	0.67
	Elbow F/E	0.95 (0.05)	10.68 (6.24)	0.27	0.70

Hand to back pocket	Shoulder F/E	0.96 (0.03)	5.14 (2.51)	0.73	0.84
	Shoulder Add/Abd	0.82 (0.18)	4.71 (2.50)	0.88	0.92
	Shoulder IR/ER	0.60 (0.23)	7.10 (2.57)	0.91	0.82
	Elbow F/E	0.89 (0.13)	8.38 (5.18)	0.85	0.88

Note: shoulder F/E, shoulder Add/Abd, shoulder IR/ER, and elbow F/E represent joint angles of shoulder flexion/extension, shoulder adduction/abduction, shoulder internal/external rotation, and elbow flexion.

## Data Availability

The data that support the findings of this study are available on request from the corresponding author, Ye Ma. The data are not publicly available yet due to the underdevelopment of the system and the ethics of the project.

## Appendix

### Appendix A: The Pearson Correlation Coefficient (*r*) of the Angles at the PTA via the Vicon and Kinect Systems

The scatterplot includes PTAs of shoulder flexion/extension (Flex/Ext), shoulder abduction/adduction (Abd/Add), shoulder internal rotation/external rotation (IR/ER), and elbow flexion/extension (Flex/Ext) during the four upper limb functional tasks. Task 1 is the hand to contralateral shoulder task. Task 2 is the hand to mouth task. Task 3 is the combing hair task. Task 4 is the hand to back pocket task. The *x*-axis of each subfigure is each individual's PTA via the Kinect system. The *y*-axis of each subfigure is each individual's corresponding PTA via the Vicon system. For each subfigure, the linear correlation between the *x*-axis value and the *y*-axis value are presented by a line and the corresponding Pearson correlation coefficient.

### Appendix B: The Pearson Correlation Coefficient (*r*) of the Range of Motion (ROM) via the Vicon and Kinect Systems

The scatterplot includes ROMs of shoulder flexion/extension (Flex/Ext), shoulder abduction/adduction (Abd/Add), shoulder internal rotation/external rotation (IR/ER), and elbow flexion/extension (Flex/Ext) during the four upper limb functional tasks. Task 1 is the hand to contralateral shoulder task. Task 2 is the hand to mouth task. Task 3 is the combing hair task. Task 4 is the hand to back pocket task. The *x*-axis of each subfigure is each individual's ROM via the Kinect system. The *y*-axis of each subfigure is each individual's corresponding ROM via the Vicon system.

### Appendix C: Scatterplots of Both the Between-Device CMCs (at Session One) and the Between-Session CMCs for the Kinect System

The left subfigure shows the between-device CMCs (session one). The right subfigure shows the between-session CMCs of Kinect system. The CMCs of shoulder flexion/extension (Flex/Ext), shoulder abduction/adduction (Abd/Add), shoulder internal rotation/external rotation (IR/ER), and elbow flexion/extension (Flex/Ext) are all presented. Four upper limb functional tasks are included: the hand to contralateral shoulder task (HCS), hand to mouth task (HM), combing hair task (CH), and hand to back pocket task (HBP).

### Appendix D: The Between-Session ICCs of the Angles at the PTA and the ROM for the Kinect System

The left subfigure shows the between-session ICCs of the angles at the PTA via the Kinect system. The right subfigure shows the between-session ICCs of the ROMs via the Kinect system. The ICCs of the angles at the PTA and the ROM in shoulder flexion/extension (Flex/Ext), shoulder abduction/adduction (Abd/Add), shoulder internal rotation/external rotation (IR/ER), and elbow flexion/extension (Flex/Ext) are all presented. Four upper limb functional tasks are included: the hand to contralateral shoulder task (HCS), hand to mouth task (HM), combing hair task (CH), and hand to back pocket task (HBP).
